# Measurement of cell kinetics in cervical tumours using bromodeoxyuridine.

**DOI:** 10.1038/bjc.1993.307

**Published:** 1993-07

**Authors:** B. S. Bolger, T. G. Cooke, R. P. Symonds, A. B. MacLean, P. D. Stanton

**Affiliations:** University Department of Surgery, Glasgow Royal Infirmary, UK.

## Abstract

The pre-treatment cell kinetics of 120 cervical tumours were assessed following the in vivo labelling with the thymidine analogue Bromodeoxyuridine (BrdUrd). In 89% both static and temporal kinetic parameters could be measured. Through the analysis of multiple biopsies from each tumour marked intra tumour heterogeneity was demonstrated. The median values for the most highly labelled sample analysed for each tumour were; S-phase duration (Ts) 12.1 h, BrdUrd labelling index (CLI) 9.5% and potential tumour doubling time 4.4 days. There was a significant elevation in CLI, but no difference in Ts, between tumour and non-neoplastic cervical tissue. There was a significant elevation in CLI, advanced stage and large size tumours. Although a significant elevation in CLI was found in aneuploid tumours this is likely to represent the systemic bias of the calculation methods, with no difference being seen between aneuploid and diploid tumours when BrdUrd labelling was measured with-out reference to the nuclei DNA content. The majority of these patients were treated with radiotherapy and cell kinetic data will be correlated with treatment response when adequate follow up has been achieved.


Br. J. Cancer (1993), 68, 166–171 © Macmillan Press Ltd., 1993

### Measurement of cell kinetics in cervical tumours using bromodeoxyuridine

B.S. Bolger[1,2], T.G. Cooke[1], R.P. Symonds[3], A.B. MacLean[2], & P.D. Stanton[1]

[1]*University Department of Surgery, Glasgow Royal Infirmary, Glasgow G31 2ER;* [2]*University Department of Midwifery, The Queen Mothers Hospital, Glasgow, G3 8SH;* [3]*University Department of Oncology, Western Infirmary, Glasgow, G11 6NT, UK.*

**Summary** The pre-treatment cell kinetcis of 120 cervical tumours were assessed following the *in vivo* labelling with the thymidine analogue Bromodeoxyuridine (BrdUrd). In 89% both static and temporal kinetic parameters could be measured. Through the analysis of multiple biopsies from each tumour marked intra tumour heterogeneity was demonstrated. The median values for the most highly labelled sample analysed for each tumour were; S-phase duration (Ts) 12.1 h, BrdUrd labelling index (CLI) 9.5% and potential tumour doubling time 4.4 days. There was a significant elevation in CLI, but no difference in Ts, between tumour and non-neoplastic cervical tissue. There was also a significant elevation in CLI in advanced stage and large size tumours. Although a significant elevation in CLI was found in aneuploid tumours this is likely to represent the systemic bias of the calculation methods, with no difference being seen between aneuploid and diploid tumours when BrdUrd labelling was measured with-out reference to the nuclei DNA content. The majority of these patients were treated with radiotherapy and cell kinetic data will be correlated with treatment response when adequate follow up has been achieved.

The rate at which a tumour proliferates is one of a number of factors important in determining the response of the tumour to cytotoxic therapy (Tubiana & Courdi, 1989). In rapidly proliferating tumours the problem of tumour repopulation between doses of radiotherapy, with resultant treatment failure, has attracted recent attention due to the potential therapeutic advantage of accelerated hyperfractionated radiotherapy (Fowler, 1986). Initial results from studies involving altered radiotherapy scheduling and measurement of cell kinetic have shown that patients with rapidly proliferating tumours may benefit from accelerated hyperfractionated schedules (Begg *et al.*, 1992). The measurement of tumour cell kinetics, particularly in tumours treated with radiotherapy, has therefore gained a new significance, with the possibility of tailoring radiotherapy schedules on the tumour proliferation characterstics.

Many methods for the measurement of tumour proliferation have been introduced over the past 30 years, which in itself indicates that no single technique developed to date has been entirely satisfactory. Early methods involved the incorporation of tritiated thymidine into newly synthesised DNA, therefore precluding their use in patients. Other approaches, including S-phase analysis from DNA histograms, assessment of proliferation associated antigens (for example Ki67) and identification of AgNORs, are only able to define static elements of proliferation, with no evaluation of phase duration. Through the incorporation of the thymidine analogue bromodeoxyuridine (BrdUrd) *in vivo*, with delayed sampling of the tumour, it is now possible to measure the duration of S-phase in addition to the fraction of proliferating cells, obtaining a more complete assessment of proliferation *in vivo* than previously feasible (Begg *et al.*, 1985). If this method of proliferation assessment is to be of clinical value it is important to determine the extent of tumour heterogeneity. This paper reports the preliminary findings of a prospective study measuring the pre treatment cell kinetics of cervical tumours using the BrdUrd incorporation technique. Due to the accessibility of cervical tumours, and the clinical feasibility of obtaining multiple biopsies, we have been able to assess the intra-tumour heterogeneity of S-phase duration and BrdUrd labelling index.

Correspondence: B.S. Bolger, University Department of Surgery, Queen Elizabeth Building, Glasgow Royal Infirmary, Alexandra Parade, Glasgow G31 2ER, UK.
Received 21 December 1992; and in revised form 1 March 1993

### Materials and methods

### *Selection of patients*

Since April 1991 all patients with cervical carcinoma scheduled for either a staging procedure prior to radiotherapy or a radical hysterectomy have been asked to give written consent for the administration of BrdUrd; 138 patients have agreed. The participating hospitals are The Beatson Oncology Centre, Stobhill Hospital, and The Royal Infirmary, Glasgow. Ethical Committee approval was given for all hospitals.

### *Bromodeoxyuridine administration*

BrdUrd was obtained from the Department of Pharmacy at the University of Strathclyde. BrdUrd 200 mg was dissolved in 100 ml of 0.9% saline and was administered intravenously over 15 min. The infusion was given 6–8 h prior to the predicted time of tumour sampling.

### *Tissue collection*

Tumour samples were collected either from radical hysterectomy specimens or by performing additional punch biopsies at the time of the staging procedure, choosing macroscopically viable areas of the tumour. When possible multiple biopsies were obtained from different areas of the tumour. If lymph node involvement was diagnosed, at time of hysterectomy, samples of an involved lymph node were also collected for kinetic evaluation. Tissue samples obtained were fixed immediately in 70% alcohol for a minimum of 24 h. In 18 cases there was no macroscopic evidence of tumour at the time of surgery. In these cases a biopsy from a part of the cervix which appeared normal was performed.

### *Tissue preparation*

A small portion of each biopsy (approximately 50 mg) was cut into 1 mm cubes using a scalpel, the remainder of the sample was processed to wax, and a 5 micron section was histologically examined to confirm the presence of tumour. The minced samples were digested enzymatically for 30 min in a 37°C agitating water bath using a 0.1% pepsin in 0.9% saline solution at pH 1.5. The digest was washed in phosphate buffered saline (PBS) and syringed with a 22 gauge needle to facilitate maximal disaggregation, before filtering the sample through a 40 micron mesh.

*BrdUrd/DNA staining*

The nuclear preparation was partially denatured with 2 M HCl for 30 min, then neutralised using 0.1 M borax. The nuclei were then washed with PBS followed by PBS containing 1% bovine serum albumin and 0.1% tween 20, prior to incubation with 0.1 ml mouse anti BrdUrd at a 1/30 dilution for 60 min. (Dako Ltd., High Wycombe). Following two washes with PBS, nuclei were incubated with 0.1 ml FITC conjugated goat anti-mouse antibody at a 1/40 dilution for 30 min. (Sigma Chemicals Ltd., Poole). Both incubations were performed at room temperature. After washing in PBS twice, the nuclei were stained with 1 ml of propidium iodide solution 10 $\mu g\ ml^{-1}$ for 30 min at room temperature.

*Flow cytometric analysis*

Samples were analysed on a Coulter Epics Profile II flow cytometer. This has a single 15 mW argon laser emitting at 488 nm. Green and red fluorescent emissions were split using a 550 nm dichroic mirror and collected through a 525 band pass and a 610 long pass filter respectively. An appropriate window was chosen from the DNA cytogram of area *vs* peak signal to eliminate debris and aggregates of nuclei. $10^4-10^5$ nuclei were analysed for each sample and the data was collected in list mode. Using the Epics Profile II software a DNA frequency histogram, a BrdUrd frequency histogram and a DNA/BrdUrd cytogram were constructed.

*Calculation of bromodeoxyuridine labelling index*

The total labelling index (TLI), representing the fraction of the entire cell population labelled with Brdu, was determined from the BrdUrd frequency histogram, the distinction between positive and negative cells was determined visually (Figure 1). A calculated labelling index (CLI%) was estimated from the BrdUrd/DNA cytogram (Figure 2), by identifying labelling associated with a specific tumour ploidy population, and by compensating for those cells which have divided since labelling (Begg *et al.*, 1985).

The CLI% is given by the equation below:

$$\text{Calculated labelling index} = \frac{\text{LuD} + [\text{LD} \times 0.5]}{\text{uL} + \text{LuD} + [\text{LD} \times 0.5]}$$

where; LuD = labelled undivided cells
LD = labelled divided cells
uL = all unlabelled cells

*Calculation of S-phase (Ts) duration*

The derivation of Ts assumes that at the time of labelling the average DNA content of labelled cells lies mid-way between the G1 and G2 peaks. It also assumes that the progression of cells through S-phase is constant. The average cell progression rate through S-phase can be calculated provided the mean DNA content of labelled undivided cells and the time interval between labelling and biopsy is known. All flash labelled S-phase cells are expected to reach G2 by a time equal to Ts, thus from the progression rate a value for Ts can be derived.

There are two circumstances where Ts could not be calculated. Firstly in some aneuploid tumours there was an overlap of the S-phase labelled cells from the diploid and aneuploid populations making calculation of the mean DNA content of labelled undivided cells impossible. Secondly, in samples with very low LI, only small numbers of labelled undivided cells were identified. If analysis produced less than 300 labelled undivided cells the sample was repeated. In practice this only proved to be a problem in samples containing no neoplastic tissue.

**Figure 1** Bromodeoxyuridine frequency histogram.

**Figure 2** DNA/BrdU cytogram (diploid tumour).

*Calculated cell kinetic parameters*

The potential doubling time was derived from the equation:

$$T_{pot} = L \frac{Ts}{CLI}$$

L is a correction factor for the non-linear distribution of cells through the cell cycle (Steel, 1977). We have used a constant value of 0.8 in our calculations.

*Analysis of multiple biopsies*

A range of results was obtained for each tumour through the analysis of multiple biopsies. The variance of results from multiple biopsies from the same tumour was compared with the variance of multiple analysis from a single biopsy. A single biopsy was enzymatically digested as above and the resultant suspension of nuclei was divided into ten aliquots. Each sample was then processed and analysed separately, two diploid and two aneuploid cell suspensions were analysed for intra assay variation, with analysis of 40 aliquots in total.

For this purpose of correlation of cell kinetic data with tumour ploidy and clinical features, and in the calculation of the summary statistics of the whole population, for each tumour the biopsy with the greatest CLI% was selected.

*Statistics*

Kinetic parameters measured do not have a normal distribution therefore the Mann Whitney test and the Kruskal Wallis test were used for comparison between two or more groups respectively. The Spearman Rank Correlation was used for comparison between tumour stage and kinetic data, and Chi-squared test for comparison of ploidy status between stages. Minitab statistical software was used for calculating this data.

### Results

A total of 138 patients with a pre-operative diagnosis of cervical carcinoma have been injected with BrdUrd. No immediate toxicity or adverse reactions have been noted. In 18 cases no neoplastic tissue was obtained at time of surgery, in 15 of these a previous diagnostic cone biopsy had removed all tumour. In two a diagnosis of primary bladder and in one of primary rectal tumour was made intra operatively. In all 18 cases a biopsy of macroscopically normal cervix was obtained and the absence of neoplastic tissue was confirmed histologically. Of the 120 tumour samples obtained, 15 were collected from patients scheduled for radical hysterectomy, and 105 at time of the staging procedure.

*Analysis of ploidy*

The mean number of samples analysed per patient was 2.8 (range 1–6), the mean number of nuclei analysed for each sample was 85,000 (range 20,000–100,000). Coefficient of variation for the G0/G1 DNA peak ranged from 2.0 to 8.2%, with a mean of 4.1%. The proportion of aneuploid and diploid tumours is shown in Figure 3a. There was no statistical significant variation in the frequency of aneuploidy with tumour stage (Chi Squared = 1.9, DF = 3, $P = 0.59$). The frequency distribution for the DNA index is shown in Figure 3b, the tumours are divided into ten groups, each group representing an intervals of DNA index of 0.2. Multiple aneuploid populations were present in six cases, in ten the DNA/BrdUrd cytogram revealed equally elevated BrdUrd labelling associated with diploid and aneuploid cell populations present, which was interpreted as representing a tumour with a mixed ploidy population. If the diploid population originated from normal stroma or lymphocytes this degree of BrdUrd would not have been expected. In seven of these 10 tumours with diploid and aneuploid elements the aneuploid population was in the tetraploid range.

**Figure 3** a, Ploidy dependent on stage b, Tumour DNA index.

*Cell kinetic parameters*

In 107 tumours (89.2%) there was adequate separation of diploid and aneuploid elements to allow calculation of S-phase duration (Ts) and calculated labelling index (CLI%), the total labelling index (TLI%) could be calculated for all tumours. Five cases where aneuploid and diploid tumour elements were present, due to the small proportion of aneuploid nuclei, the diploid element was analysed in the assessment of Ts and CLI%. Twelve of the eighteen cases where samples did not contain neoplastic tissues Ts could not be calculated due to inadequate numbers of labelled undivided nuclei. The median values and quartile range for kinetic parameters in tumour and non-neoplastic samples are shown in Table I. There is a significant elevation in BrdUrd labelling in tumour samples but no difference in Ts ($P = 0.0001$ and $P = 0.72$ respectively).

*Intra tumour heterogeneity*

The variation in cell kinetic parameters measured between multiple biopsies from the same tumour was assessed for those tumours where three or more biopsies were analysed and in polyploid tumours where the ploidy of the analysed population was the same in all samples. These criteria were satisfied in 21 aneuploid and 22 diploid tumours, a mean of 3.6 biopsies per patient were analysed, (range 3–6). Each individual measurement for the tumour is expressed as a fraction of the mean for this tumour. In the assessment of intra assay variation each result was expressed as a ratio of the mean for the ten aliquots analysed. The variation in Ts and CLI% measurement seen between multiple biopsies of the same tumour can not be explained by intra assay variation and therefore represent intra tumour heterogeneity, (Table II). The variation in CLI% is greater than variation in Ts measurement.

*Cell kinetics and tumour ploidy*

A significant elevation in CLI% is seen in aneuploid tumours ($P = 0.002$) but there is no difference in TLI or Ts, (Table IV). There is however systematic bias in the measurement of CLI% between diploid and aneuploid tumours due to the admixture of normal cells with diploid tumours only. TLI% measurement, although less precise, does not introduce this bias. No difference in TLI between ploidy groups is seen which suggests that for cervical tumours there is no increase in BrdUrd labelling an aneuploid tumours.

*Cell kinetics and clinical parameters*

A progressive elevation in CLI% is seen with advancing stage (Spearman Rank Correlation $r = 0.27$, $P = .005$), no variation in Ts was noted (Figures 4 and 5). A significant decrease in the Tpot. was seen with increasing stage (Spearman Rank Correlation $r = 0.27$, $P = .005$). A similar increase in TLI with tumour stage was also seen ($r = 0.24$, $P = 0.008$).

**Table I** Cell kinetics of tumour and non-neoplastic tissue

| | *Median Ts hours* | *Median CLI %* | *Median Tpot days* |
|---|---|---|---|
| Tumour $n = 107$ | 12.1 | 9.5 | 4.4 |
| (Inter Quartile Range) | (10.7–14.7) | (6.2–14.7) | (3.1–6.4) |
| Non-neoplastic $n = 6$ | 12.2 | 0.42 | 43.3 |
| (Inter Quartile Range) | (9.6–14.3) | (0.2–0.8) | (27.3–86.6) |
| *P* value | 0.72 | 0.0001 | 0.0001 |
| 95% C.I. | – 1.9 to 3.6 | 5.0 to 12.1 | 17.0 to 39.2 |

**Table II** Intra tumour heterogeneity

| | *Standard deviation of* | *Individual measurement / mean for tumour* | |
|---|---|---|---|
| | *Ts* | *CLI%* | *Tpot.* |
| *Tumour samples* | | | |
| Aneuploid $n = 76$ (21 tumours) | 14.0% | 19.8% | 18.4% |
| Diploid $n = 80$ (22 tumours) | 12.7% | 30.0% | 34.2% |
| *Intra Assay Variation* | | | |
| Aneuploid $n = 20$ (2 tumours) | 3.1% | 2.6% | 5.2% |
| Diploid $n = 20$ (2 tumours) | 1.8% | 2.5% | 3.5% |

**Table III** Cell kinetics and clinical parameters

| | *Median Ts hours* | *Median CLI %* | *Median Tpot. days* |
|---|---|---|---|
| *Size* | | | |
| $<5$ cm $n = 55$ | 11.7 | 9.0 | 4.9 |
| $>5$ cm $n = 52$ | 12.5 | 11.7 | 4.0 |
| *P* value | 0.15 | 0.04 | 0.08 |
| 95% C.I. | – 1.9 to 0.3 | 0.1 to 4.4 | – 0.01 to 1.6 |
| *Lymph node status* | | | |
| positive $n = 4$ | 10.1 | 6 | 6.7 |
| negative $n = 11$ | 12.6 | 6.6 | 6.3 |
| *P* value | 0.13 | 0.95 | 0.56 |
| 95% C.I. | – 1.5 to 10.4 | – 5.2 to 7.1 | – 3.1 to 9.2 |
| *Age* | | | |
| $>55$ ($n = 50$) | 12.4 | 9.4 | 4.5 |
| 45–55 ($n = 14$) | 12.4 | 11.7 | 4.0 |
| $<45$ ($n = 14$) | 11.8 | 8.6 | 3.8 |
| *P* value | 0.14 | 0.51 | 0.55 |

An increase in CLI% is also observed in tumours of $>5$ cm size compared with tumours $<5$ cm ($P = .04$), but again no difference in Ts was noted (Table III). To determine the effect of patients age on tumour cell kinetics all women with stage 2B, or more advanced tumours, were analysed following division into three age groups, $<45$, 45–55, and $>55$. No difference is seen in any of the kinetic parameters measured between these groups, (Table III).

The relationship between the cell kinetics of the primary tumour and the chance of metastatic disease was analysed in the group of patients who were scheduled for a radical hysterectomy and pelvic lymphadenectomy. Four out of fifteen had histologically proven lymph node metastatic deposits, in three of these four, biopsies from primary and metastatic tumour were obtained. There was no significant difference in the primary tumour cell kinetics dependent on the presence of lymph node involvement, (Table III). Three of four tumours with lymphatic involvement were diploid, and ploidy of primary and secondary tumour was the same in all cases. No consistent difference in kinetic parameters was seen between the primary and secondary tumour.

### Discussion

The study of human tumour cell kinetics has been facilitated by the use of halogenated pyrimidines to label proliferating cells. In addition to the advantage of being able to safely label tumours *in vivo*, measurement of cell cycle phase duration (Ts) and calculation of Tpot, is now feasible. In a recent review of over 600 tumours labelled with BrdUrd, variation in Ts was almost as great as variation in LI, suggesting that it may be an important parameter (Wilson, 1991). Due to the relatively recent introduction of these techniques into clinical studies little data are available at present to correlate the temporal and static proliferation parameters measured with long term survival.

A number of studies have evaluated proliferation status in

**Figure 4** Variation in labelling index with clinical stage.

**Figure 5** Variation in S-phase duration with clinical stage.

Table IV Cell kinetics and tumour ploidy

| | Median Ts hours | Median CLI % | Median TLI % | Median Tpot. days |
|---|---|---|---|---|
| Tumour ploidy | | | | |
| Aneuploid $n = 67$ | 12.5 | 11.6 | 8.6 | 4.0 |
| Diploid $n = 40$ | 11.8 | 7.4 | 7.8 | 5.3 |
| $P$ value | 0.27 | 0.002 | 0.61 | 0.01 |
| 95% C.I. | −0.5 to 1.8 | 1.2 to 5.4 | −2 to 1.4 | 0.25 to 2.1 |

cervical tumours with conflicting results regarding the correlation of proliferation status to survival or recurrence rate. Two have reported an increased short term recurrence rate in tumours with high S-phase rates, whereas two others did not confirm this effect in tumours with a high tritiated thymidine labelling or in tumours with elevated Ki67 expression (Strang *et al.*, 1987; Naus & Zimmerman, 1991; Dixon *et al.*, 1977; Cole *et al.*, 1992). None of these studies however have adequately addressed the problem of intra tumour heterogeneity or described the range of values obtained by multiple sampling of tumours. Our assessment of static and temporal kinetic parameters, performed *in vivo*, with analysis of multiple biopsies gives a more complete assessment of cervical tumour proliferation than any previous study.

These preliminary results demonstrate that measurement of cell kinetics using BrdUrd is feasible in cervical tumours. In all cases studied a value for total BrdUrd labelling (TLI) could be calculated, and in 89% both temporal and static parameters could be assessed. Cervical tumours do however show marked intra tumour heterogeneity. In aneuploid tumour, where analysis should not be affected by varying proportions of normal tissue contamination, a wide spread of values for CLI and Ts were obtained from multiple samples from the same tumour. The difference is variation in measured CLI% between diploid and aneuploid tumours is likely to represent the inability of this technique to differentiate normal from tumour cells in diploid tumours, rather than representing greater heterogeneity in diploid tumours. The degree of variation in cell kinetic seen in this study emphasises that inadequacies of proliferation studies based on the analysis of single samples and may be a major limitation in the clinical application of results obtained from tumour proliferation studies.

Values for Ts, LI and Tpot obtained in this study are comparable with results obtained by Bennington following *in vivo* labelling with tritiated thymidine and Wilson *et al.*, using similar BrdUrd incorporation techniques (Wilson, 1991; Bennington, 1969). The wide range of potential tumour doubling times observed amphasises the possible advantages for the treatment of cervical tumours with accelerated or hyperfractionated radiotherapy schedules, provided an accurate representative assessment of tumour proliferation can be made.

The elevation in LI in advanced stage disease and in large tumours seen in this study, may occur as a result of evolution of the tumour cell kinetics as progression occurs. Another possible interpretation of these findings is that clinical stage at diagnosis is related to the biological properties of the tumour, with advanced stage, large tumours representing a more proliferative group.

The presence of aneuploid cell populations in a number of tumour types is associated with higher LI and poor prognosis (Tribukait, 1984; Dressler *et al.*, 1988). The comparison of CLI% between aneuploid and diploid tumours is not valid because of the inherent systematic bias due to the inclusion of non-tumour cells within diploid samples. The similar TLI seen in both diploid and aneuploid tumours indicates that there is no difference in cell proliferation between ploidy types. This conclusion has been supported by the finding of a simular median BrdUrd labelling index determined from immunohistochemical staining of tissue sections for diploid and aneuploid tumours (unpublished data). The apparent lack of proliferative advantage in aneuploid cervical tumours may explain the variable results obtained when comparing ploidy with survival in this tumour type, with a number of studies failing to show a survival advantage for a particular ploidy group (Hendy-Ibis *et al.*, 1987; Davis *et al.*, 1989; Kenter *et al.*, 1990).

Prolongation of S-phase has been noted in cancer cells (Steel, 1977). Comparison of tumour with non-neoplastic tissues in this study showed no alteration in Ts. The majority of non neoplastic cervical tissue analysed in this study originated from cervices in which a previous diagnostic biopsy had removed all tumour (15/18), and in addition in only 1/3 of these samples could values for Ts and CLI be calculated, these results therefore may not be representative of the whole group. The value for kinetic parameters obtained may therefore not accurately reflect that of normal cervix. By obtaining cervical biopsies from patients with ovarian tumours who have received BrdUrd we hope to obtain a more confident assessment of normal cervical cell kinetics.

The determination of tumour ploidy is facilitated by analysis of the BrdUrd/DNA cytogram. A group of patients with tumours in which both diploid and aneuploid tumour elements exist may be identified. The existence of this ploidy variation has been noted previously in tumour karyotype studies (Tribukait, 1984). If the diploid tumour cells only constitute a small proportion of the tumour cells present, or if the diploid tumour proliferation rate is low, the BrdUrd/DNA cytogram will be unable to detect the presence of diploid tumour element. The frequency of combined aneuploid diploid tumours is therefore likely to be higher than the 8% measured here. This ploidy variation is frequently not reported in simple DNA histogram analysis.

The prognostic powers of kinetic data calculated using BrdUrd incorporation techniques can only be accurately assessed when clinical follow-up data are available. Cell kinetic data will only be of prognostic value if it can predict prognosis independently of existing known prognostic variables. Comparison of the data obtained with accepted prognotic indicators is therefore of limited value. We have presented a basic analysis of the data with no attempt to correct for associations between stage, size and age. The proportion of tumour cells actively proliferating, but not S-phase duration, appears to be related to the local growth of the tumour, with a significant difference in LI, but not Ts, being seen between tumour stages and between small and large tumours. Cell kinetics of the primary tumour however appear unrelated to the incidence of lymphatic involvement, with no difference in kinetic parameters measured, dependant on the presence of lymph node involvement at the time of radical hysterectomy and pelvic lymphadenectomy. The debate regarding the possible poor prognosis of young women with cervical tumours remains inconclusive (Peel *et al.*, 1991). This study was unable to show a difference in kinetic parameters dependent on age. We do intend to compare single pathological review of all tumours with kinetic parameters measured.

For advanced tumours, in addition to receiving conventional radiotherapy, where appropriate, patients are entered into a phase three study of neo-adjuvant chemotherapy. Analysis of radiotherapy and chemotherapy response in relation to pre treatment tumour cell kinetics will therefore be available in the next few years when adequate follow-up data has been collected. We will also be able to assess the relative importance of mean and maximum values obtained from multiple tumour samples and hope to be able to provide clinical guidelines relating to optimal cervical tumour sampling for the measurement of tumour cell kinetics.

This study has been funded by the Scottish Home and Health department. We would like to thank Drs Davis, Kennedy, Habeshaw and Reid for allowing their patients to be recruited into the study and for obtaining the tumour biopsies, and to acknowledge the help of Dr G Murray for his statistical advice.

### References

BEGG, A.C., HOFLAND, I., VAN GLABEKKE, M., BARTLINK, H. & HORIOT, J.C. (1992). Predictive value of potential doubling time for radiotherapy of head and neck tumour patients. *Sem. Radiat. Oncol.* , **2,** 22-25.

BEGG, A.C., MCNALLY, N.J., SHRIEVE, D.C. & KARCHER, H. (1985). A method to measure the duration of DNA synthesis and the potential doubling time from a single sample. *Cytometry*, **6,** 620-626.

BENNINGTON, J.L. (1969). Cellular kinetics of invasive squamous carcinoma of the human cervix. *Cancer Res.*, **29,** 1082-1088.

COLE, D.J., BROWN, D.C., CROSSLEY, E., ALCOCK, C.J. & GATTER, K.C. (1992). Carcinoma of the cervix uteri; an assessment of the relationship of tumour proliferation to prognosis. *Br. J. Cancer*, **65,** 783-785.

DAVIS, J.R., ARISTIZABEL, S., WAY, D.A., WEINER, S.A., HICKS, M.J. & HAGAMAN, R.M. (1989). DNA ploidy, grade and stage in the prognosis of uterine cervical cancer. *Gynecol. Oncol.*, **32,** 4-7.

DIXON, B., WARD, A.J. & JOSLIN, C.A.F. (1977). Pre-treatment tritiated thymidine labelling of cervical biopsies; histology, staging, and tumour response to radiotherapy. *Clin. Radiol.*, **28,** 491-497.

DRESSLER, L.G., SEAMER, L.C., OWENS, M.A., CLARK, G.M. & MCGUIRE, W.L. (1988). DNA flow cytometry and prognostic factors in 1331 frozen breast cancer specimens. *Cancer*, **61,** 420-427.

FOWLER, J.F. (1986). Potential for increasing the differential response between tumours and normal tissues: can proliferation rate be used? *Int. J. Radiat. Oncol, Biol. Phys.*, **12,** 641-645.

HENDY-IBIS, P., COX, H., EVAN, G.I. & WATSON, J.V. (1987). Flow cytometric quantitation of DNA c-myc oncoprotein in archival biopsies of the uterine cervix neoplasia. *Br. J. Cancer*, **55,** 275-282.

KENTER, G.G., CORNELISSE, C.J., AARTSEN, E.J., MOOY, W., HERMANS, J., HEINTZ, A.P. & FLEUREN, G.J. (1990). DNA ploidy level in low stage carcinoma of the uterine cervix. *Gynecol. Oncol.*, **39,** 181-185.

NAUS, G.J. & ZIMMERMAN, R.L. (1991). Prognostic value of flow cytophotometric DNA content analysis in single treatment stage IB-IIA squamous cell carcinoma of the cervix. *Gynecol-Oncol.*, **43,** 149-153.

PEEL, K.R., KHOURY, G.G., JOSLIN, C.A.F., O'DONOVAN, P.J., MGAYA, H., KEATES, G., HEAD, C. & THOROGOOD, D.J. (1991). Cancer of the cervix in women under 40 years of age, a regional survey, 1975-1984. *Br. J. Obstet. Gynaecol.*, **98,** 993-100.

STEEL, G.G. *Growth Kinetics of Tumours*, Oxford: Clarendon Press, 1977.

STRANG, P., ELKUND, G., STANDHL, U. & FRANFENDAL, B. (1987). S-phase rate as a predictor of early recurrence in carcinoma of the uterine cervix. *Anticancer. Res.*, **7,** 807-810.

TRIBUKAIT, B. (1984). Clinical DNA flow cytometry. *Mel. Oncol. & Tumour Pharmacother.*, **1,** 211-218.

TUBIANA, M. & COURDI, A. (1989). Cell proliferation kinetics in human solid tumours: relation to probability of metastatic dissemination and long term survival. *Radiother. Oncol.*, **15,** 1-18.

WILSON, G.D. (1991). Assessment of human tumour proliferation using bromodeoxyuridine-current status. *Acta. Oncol.*, **30,** 903-910.


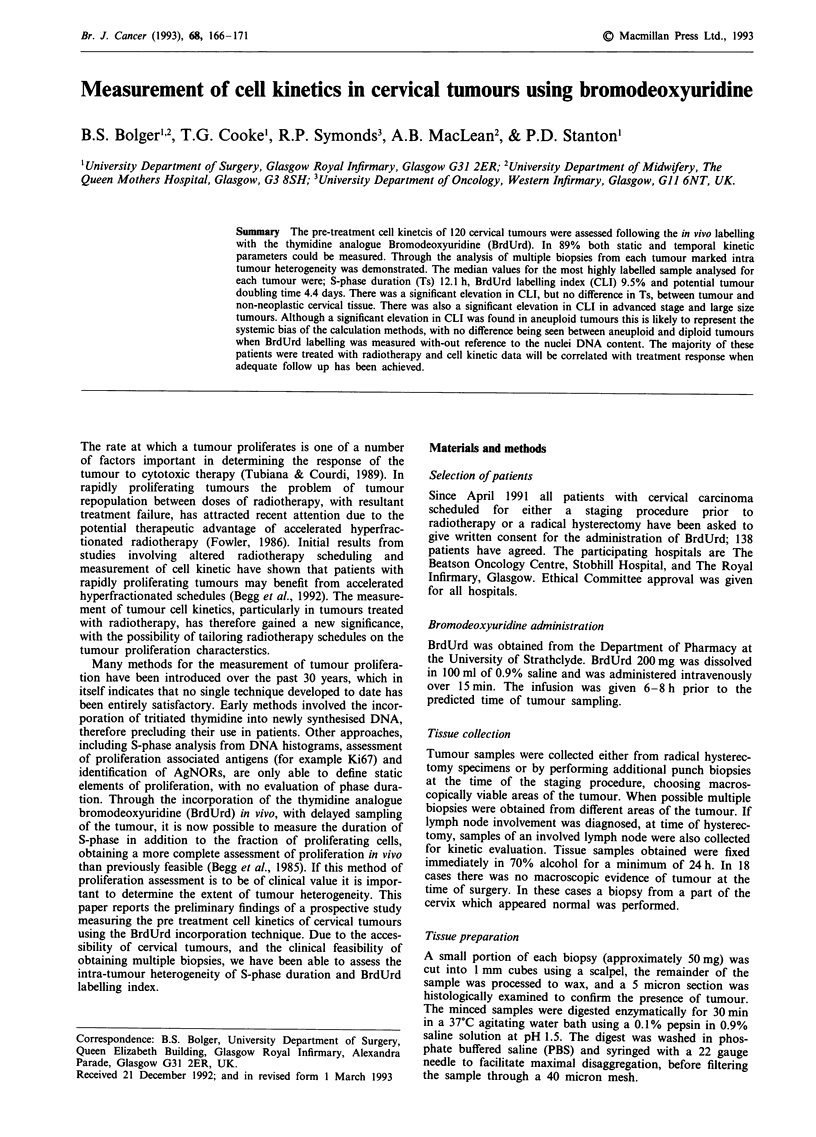

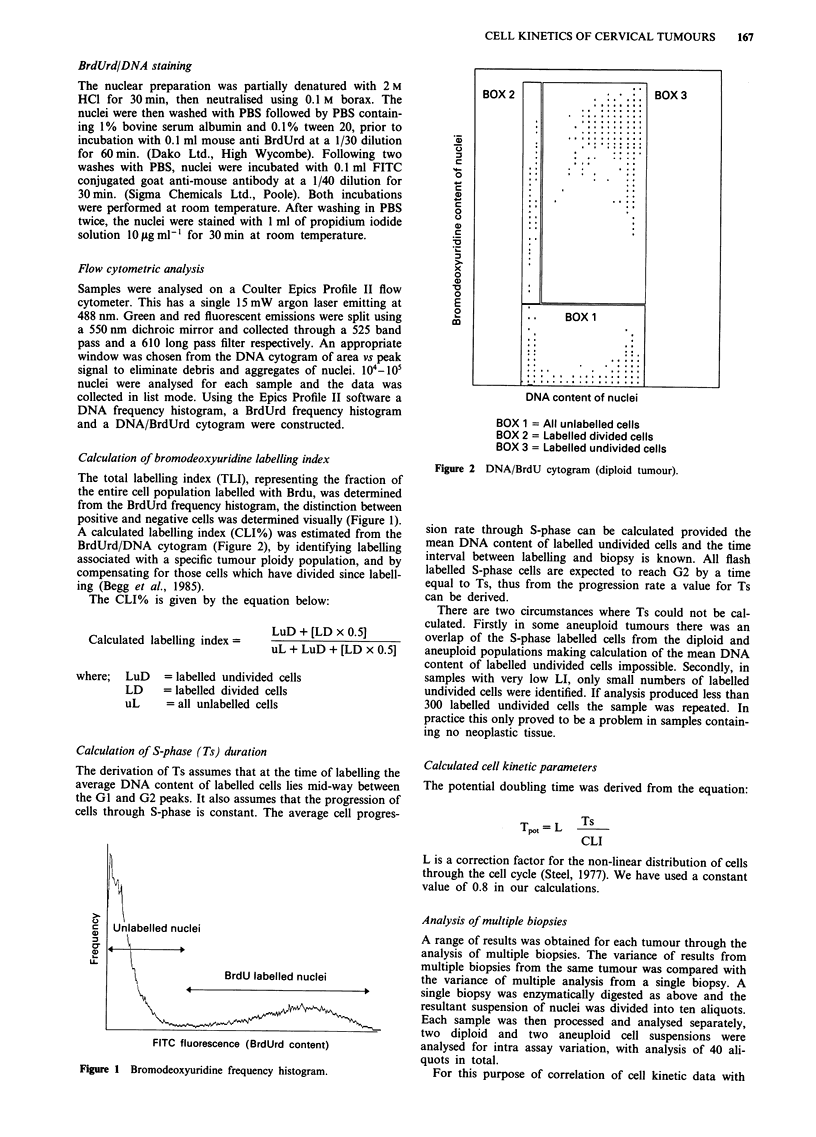

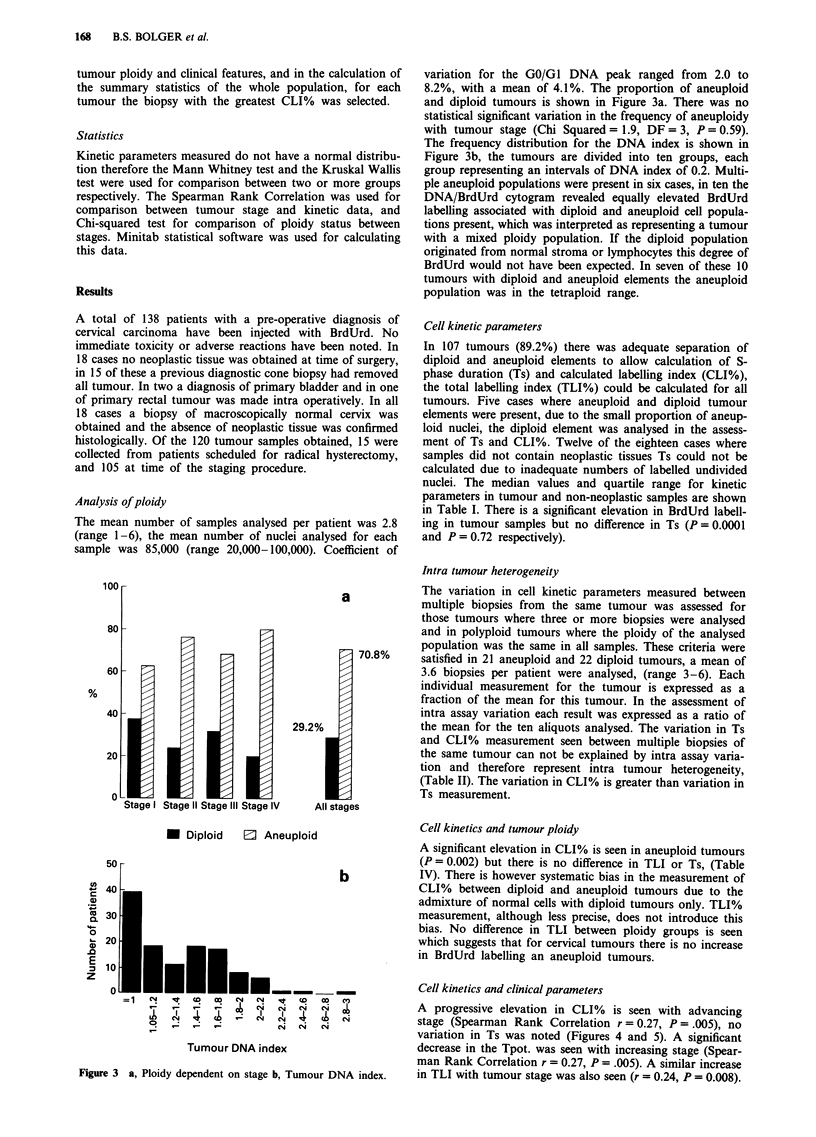

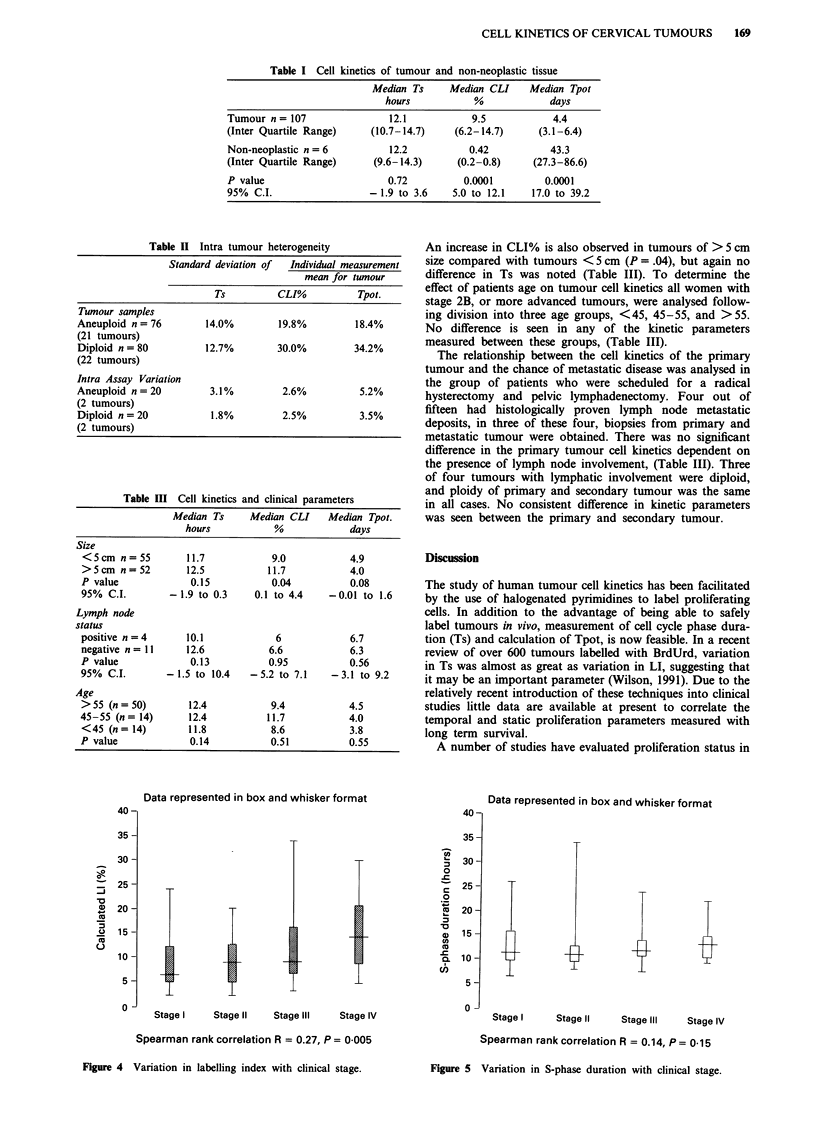

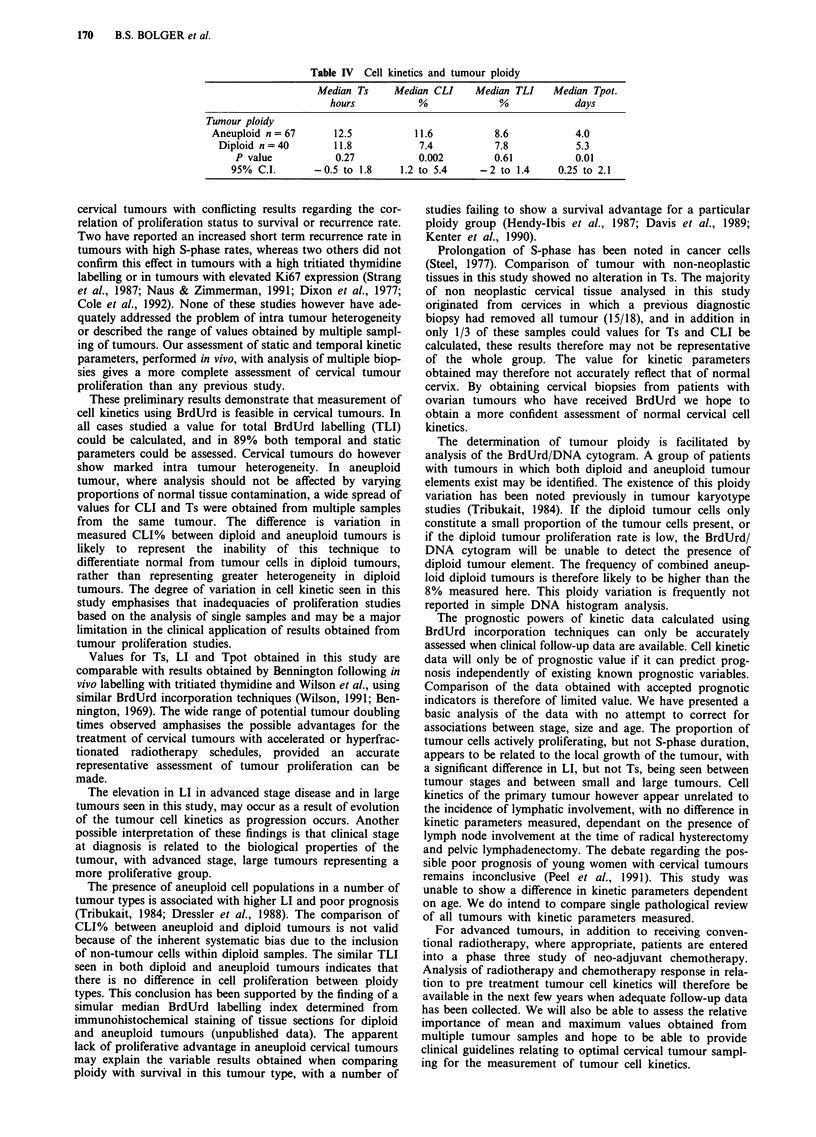

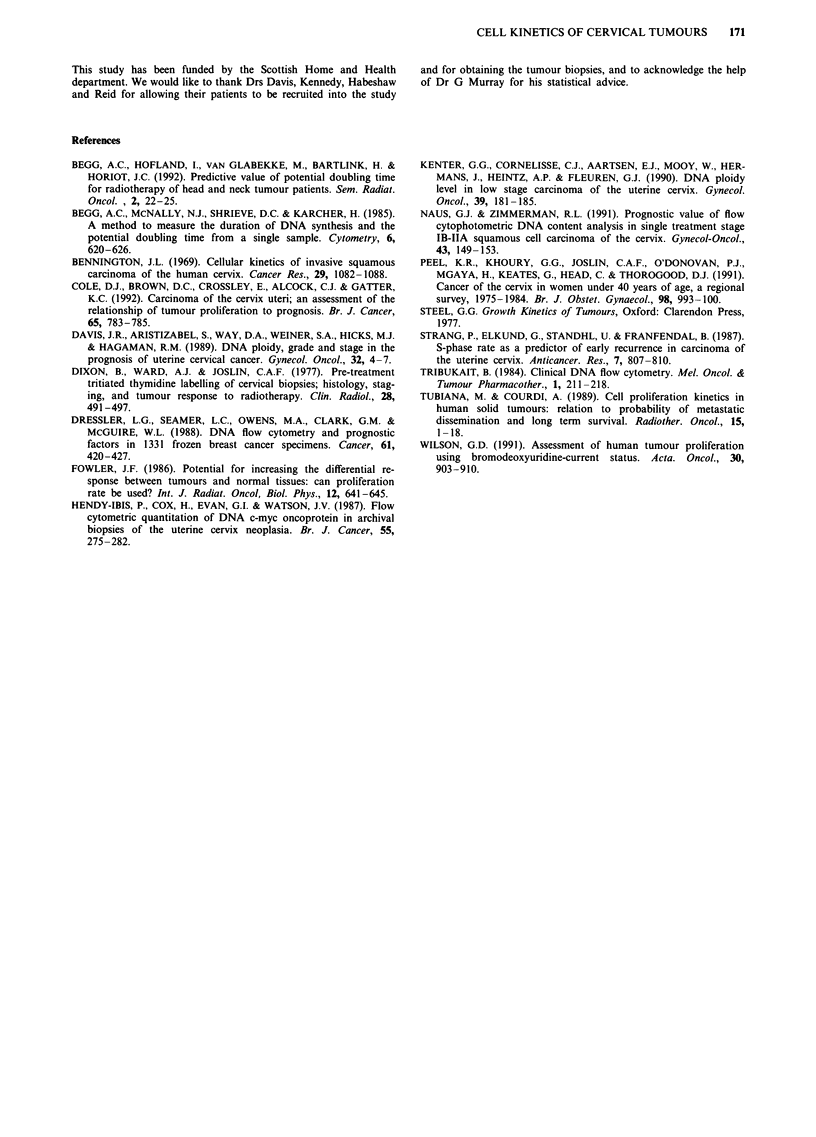


## References

[OCR_00827] Begg A. C., McNally N. J., Shrieve D. C., Kärcher H. (1985). A method to measure the duration of DNA synthesis and the potential doubling time from a single sample.. Cytometry.

[OCR_00833] Bennington J. L. (1969). Cellular kinetics of invasive squamous carcinoma of the human cervix.. Cancer Res.

[OCR_00837] Cole D. J., Brown D. C., Crossley E., Alcock C. J., Gatter K. C. (1992). Carcinoma of the cervix uteri: an assessment of the relationship of tumour proliferation to prognosis.. Br J Cancer.

[OCR_00843] Davis J. R., Aristizabal S., Way D. L., Weiner S. A., Hicks M. J., Hagaman R. M. (1989). DNA ploidy, grade, and stage in prognosis of uterine cervical cancer.. Gynecol Oncol.

[OCR_00848] Dixon B., Ward A. J., Joslin C. A. (1977). Pre-treatment 3H-TdR labelling of cervical biopsies: histology, staging and tumour response to radiotherapy.. Clin Radiol.

[OCR_00854] Dressler L. G., Seamer L. C., Owens M. A., Clark G. M., McGuire W. L. (1988). DNA flow cytometry and prognostic factors in 1331 frozen breast cancer specimens.. Cancer.

[OCR_00865] Hendy-Ibbs P., Cox H., Evan G. I., Watson J. V. (1987). Flow cytometric quantitation of DNA and c-myc oncoprotein in archival biopsies of uterine cervix neoplasia.. Br J Cancer.

[OCR_00873] Kenter G. G., Cornelisse C. J., Aartsen E. J., Mooy W., Hermans J., Heintz A. P., Fleuren G. J. (1990). DNA ploidy level as prognostic factor in low stage carcinoma of the uterine cervix.. Gynecol Oncol.

[OCR_00877] Naus G. J., Zimmerman R. L. (1991). Prognostic value of flow cytophotometric DNA content analysis in single treatment stage IB-IIA squamous cell carcinoma of the cervix.. Gynecol Oncol.

[OCR_00883] Peel K. R., Khoury G. G., Joslin C. A., O'Donovan P. J., Mgaya H., Keates G., Head C., Thorogood D. J. (1991). Cancer of the cervix in women under 40 years of age, a regional survey, 1975-1984.. Br J Obstet Gynaecol.

[OCR_00893] Strang P., Eklund G., Stendahl U., Frankendal B. (1987). S-phase rate as a predictor of early recurrences in carcinoma of the uterine cervix.. Anticancer Res.

[OCR_00898] Tribukait B. (1984). Clinical DNA flow cytometry.. Med Oncol Tumor Pharmacother.

[OCR_00902] Tubiana M., Courdi A. (1989). Cell proliferation kinetics in human solid tumors: relation to probability of metastatic dissemination and long-term survival.. Radiother Oncol.

[OCR_00908] Wilson G. D. (1991). Assessment of human tumour proliferation using bromodeoxyuridine--current status.. Acta Oncol.

